# Carcinomatous Meningitis and Hydrocephalus in Plasmacytoid Urothelial Carcinoma of the Urinary Bladder With Extremely Elevated CA19-9 Levels

**DOI:** 10.7759/cureus.54643

**Published:** 2024-02-21

**Authors:** Fumiaki Henmi, Kayako Ukai, Atsuhito Nakayama, Yutaka Takazawa, Yoshikazu Uesaka

**Affiliations:** 1 Department of Neurology, Toranomon Hospital, Tokyo, JPN; 2 Department of Pathology, The University of Tokyo, Tokyo, JPN; 3 Department of Pathology, Toranomon Hospital, Tokyo, JPN

**Keywords:** span-1, leptomeningeal metastasis, bladder cancer, leptomeningeal carcinomatosis, urothelial carcinoma, ca19-9, hydrocephalus, carcinomatous meningitis, plasmacytoid urothelial carcinoma

## Abstract

This case report describes a rare and aggressive presentation of plasmacytoid urothelial carcinoma (PUC) with carcinomatous meningitis, hydrocephalus, extensive organ involvement, and extremely elevated serum CA19-9 levels. Autopsy findings revealed that PUC of the urinary bladder origin caused carcinomatous meningitis and hydrocephalus, with exacerbation of hydrocephalus as the direct cause of death. Immunohistochemical studies confirmed the bladder origin of PUC, and PUC cells were positive for CA19-9, a tumor marker commonly associated with gastrointestinal malignancies, suggesting that the markedly high serum CA19-9 level was related to the tumor-producing mechanism.

## Introduction

Carcinomatous meningitis, also referred to as "leptomeningeal metastasis" or "leptomeningeal carcinomatosis," is an infrequent complication observed in various malignancies such as breast, lung, melanoma, gastrointestinal, and primary central nervous system tumors, but is rarely reported in urothelial carcinomas.

Plasmacytoid urothelial carcinoma (PUC) is a rare histologic subtype representing approximately 2% of invasive bladder cancers [1,2], first described by Sahin et al. in 1991 [3]. This subtype is diffusely invasive, often at an advanced stage at the time of detection, and has an aggressive clinical course and poor prognosis.

Currently, no blood tumor marker has been identified for urothelial carcinomas. Serum CA19-9 is highly positive in gastrointestinal cancers such as pancreatic cancer, gallbladder cancer, and cholangiocarcinoma, but there have been few reports in cases of PUC. Case reports of carcinomatous meningitis caused by PUC are rare, and no previous reports have indicated the production of CA19-9 by PUC.

In this report, we present an autopsy case of bladder cancer with elevated serum CA19-9 levels that resulted in carcinomatous meningitis and hydrocephalus, accompanied by a brief review of the literature.

## Case presentation

A 73-year-old male with a past medical history of essential hypertension, diabetes mellitus type 2, and chronic kidney disease presented with anorexia, nausea, and difficulty walking. He had no notable social or family history. Ten days prior to admission, he began to experience epigastric pain and anorexia, consuming only a small breakfast and light meals. Gradually, his fatigue increased, making it challenging for him to move. Two days before admission, he fell down several times a day and consulted our neurology department. A brain MRI revealed hydrocephalus, and the patient was admitted to our hospital for further assessment.

The patient's blood pressure was 98/66 mmHg, pulse rate 87 beats per minute, body temperature 35.9°C, and oxygen saturation 96% on room air. General physical examination showed diffuse abdominal pain and mild tenderness in the lower abdomen. Neurological examination revealed mild consciousness disturbance (JCS I-2, GCS E4V4M6). Speech was slow, and there were difficulties in following instructions, but no clear signs of cranial nerve deficit or limb paralysis were observed. Kernig's sign and Lasegue's sign were positive. While the patient was originally able to ride a bicycle, he had become unable to walk independently and required assistance for transferring to a wheelchair.

**Figure 1:**
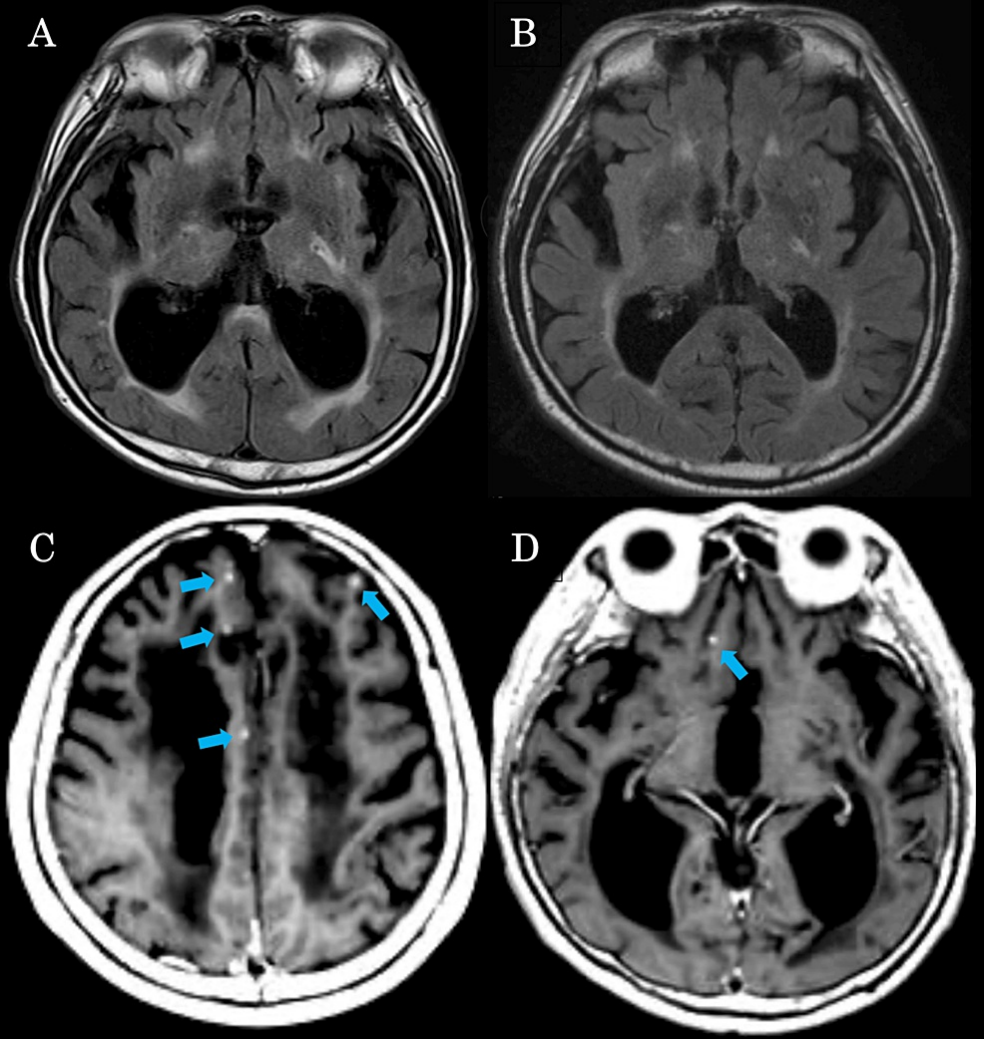
Plane brain MRI and contrast-enhanced MRI findings upon admission. Axial FLAIR brain MRI on admission shows enlarged bilateral ventricles (A) compared with axial FLAIR brain MRI seven years ago (B). Axial T1-weighted brain contrast-enhanced MRI shows multiple enhancement lesions on the brain sulci (C, D arrows). FLAIR: fluid-attenuated inversion recovery.

Blood tests showed a normal complete blood count, normal inflammation markers (CRP 0.23 mg/dL), abnormalities in electrolytes (potassium concentration was 5.4 mEq/L), elevated creatinine (1.7 mg/dL), and increased D-dimer (3.7 μg/mL). Liver function was normal. Cerebrospinal fluid (CSF) analysis revealed elevated pressure (275 mmH₂O) and pleocytosis (37/µL) with predominantly mononuclear cells (36 mononuclear cells, 1 polymorphonuclear cell), and glucose level was at 79 mg/dL (blood glucose level was at 149 mg/dL), and normal protein level (35 mg/dL), and IgG index was mildly elevated to 0.87. CSF culture was negative, and PCR tests for herpes simplex virus DNA and varicella-zoster virus DNA were negative. CSF cytology was classified as Class III. A brain MRI scan demonstrated significant symmetrical enlargement of the lateral ventricles compared to the previous study seven years ago (Figure 1A,B). Gadolinium-enhanced T1-weighted images revealed multiple enhancing lesions in the brain parenchyma and sulci (Figure 1C,D). Lumbar spine MRI scan did not show contrast enhancement in the spinal cord or cauda equina.

**Figure 2:**
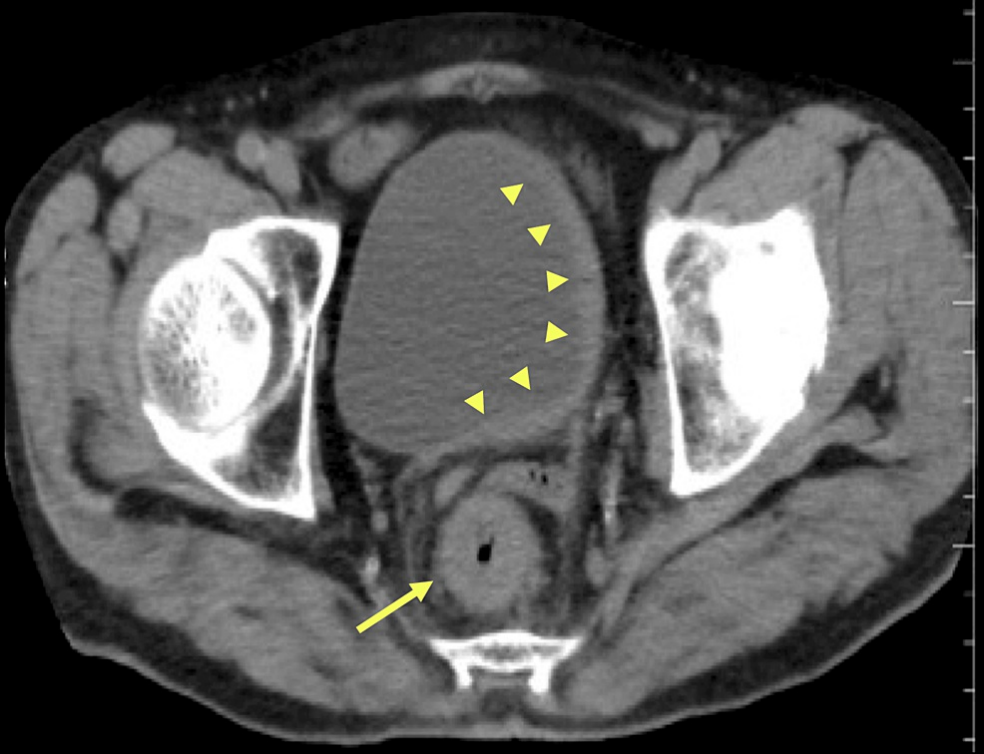
Abdominal contrast-enhanced CT findings. Asymmetric thickening of the bladder wall and rectal wall was observed on abdominal contrast-enhanced CT.

Due to persistent anorexia, vomiting, and abdominal pain, an abdominal CT was performed, which revealed thickening of the left bladder wall and circumferential thickening of the rectum (Fig. 2). Tumor markers in serum were significantly elevated, with carbohydrate antigen (CA) 19-9 at 3378 U/mL (normal range 0-37 U/mL) and SPan-1 at 1720 U/mL (normal range 0-30 U/mL), whereas levels of carcinoembryonic antigen (CEA) and prostate-specific antigen (PSA) were normal.

**Figure 3:**
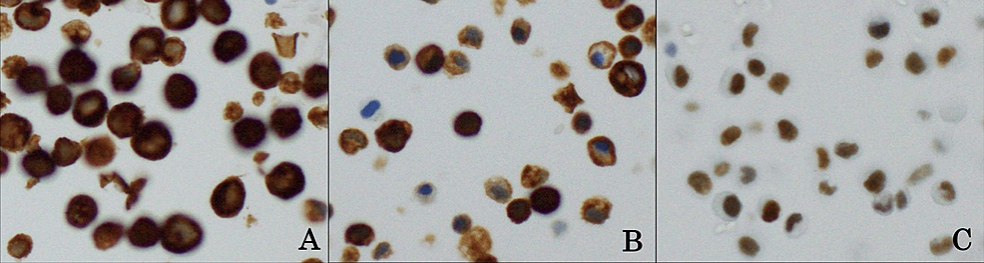
Immunostain of CSF cell block preparation. Atypical cells are positive for CK7 (A), CK20 (B), and GATA3 (C).

Additional imaging studies, including abdominal contrast-enhanced CT, abdominal ultrasound, and magnetic resonance cholangiopancreatography (MRCP), did not show definitive tumor lesions in the pancreas or biliary system. Diffusion-weighted imaging (DWI) on MRI revealed multiple hyperintense lesions in various vertebrae, suggesting extensive bone metastasis. Urine cytology indicated Class IV, suggesting suspicion of urothelial carcinoma. Cystoscopy revealed papillary tumors and irregular mucosa in the bladder wall, but a biopsy was not performed for a definitive diagnosis. Based on the cytology and imaging findings, carcinomatous meningitis due to invasive bladder cancer with multiple bone metastases was suspected. Tumor markers continued to rise, reaching CA19-9 27,359 U/mL and SPan-1 5,765 U/mL. CSF CA19-9 was elevated at 67 U/mL (normal range 0-32 U/mL). CSF cytology was performed four times (days 1, 4, 20, 27), resulting in a Class IV diagnosis, showing a loosely cohesive epithelial tumor with a ring-like cell appearance. In the immunostain of CSF cell block preparation, atypical cells are positive for CK7 (A), CK20 (B), and GATA3 (C) (Figure 3).

Subsequently, the patient's consciousness gradually worsened, and a head CT revealed worsening hydrocephalus. Considering the patient's condition, chemotherapy was deemed inappropriate, and the patient was treated with only palliative therapy. From day 60 of hospitalization, the patient developed Cheyne-Stokes respiration, and eventually, the heart rate and respiratory rate declined, and he died 63 days after admission. An autopsy was performed on the same day (postmortem, 9 hours).

**Figure 4:**
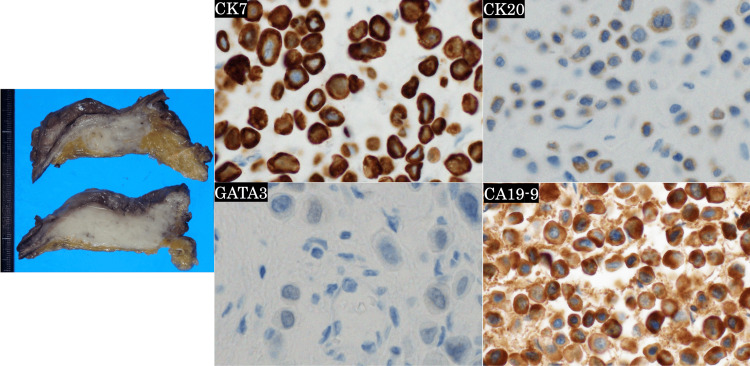
Pathological findings of the urinary bladder carcinoma in autopsy. Macroscopically marked thickening of the urinary bladder wall was observed. Immunohistochemically, the tumor was positive for CK7, CK20, GATA3 (weakly), and CA19-9.

Autopsy revealed marked thickening of the bladder wall (Figure 4). Immunohistochemically, the urinary bladder wall tumor was positive for CK7, CK20, and GATA3, which is consistent with the PUC subtype. Additionally, immunostaining for CA19-9 was positive (Figure 4).

**Figure 5:**
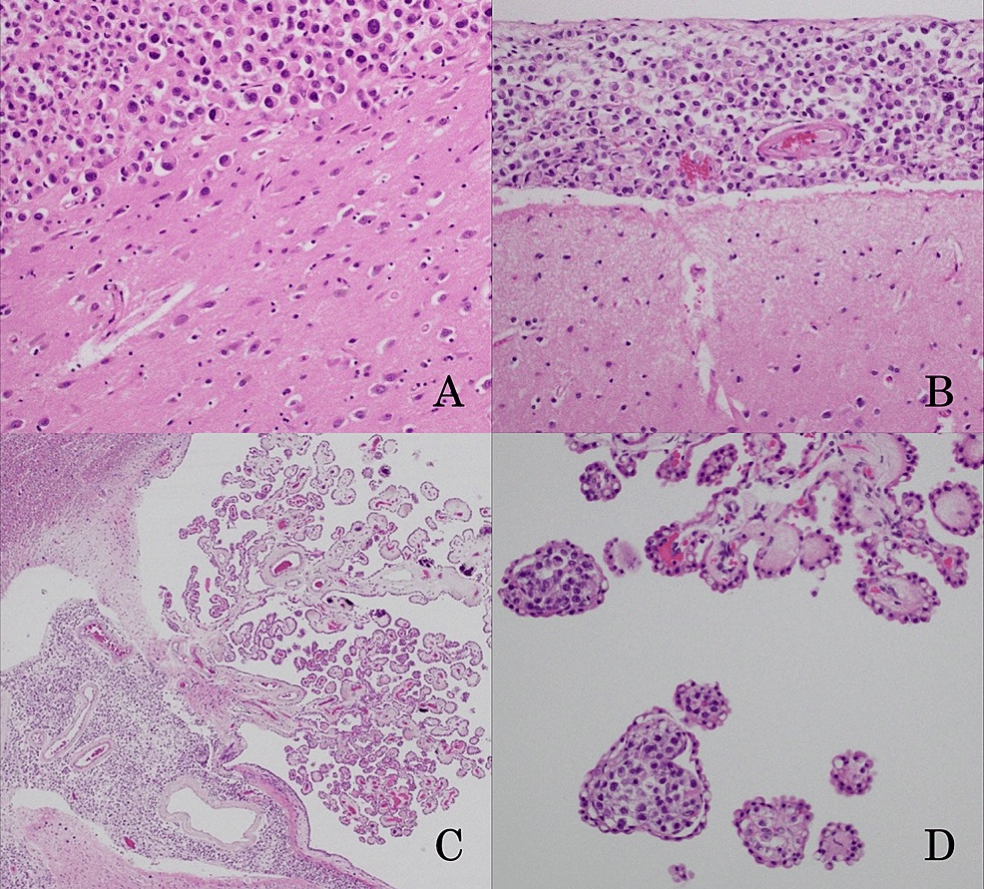
Histopathological findings of the central nervous system at autopsy. Metastatic carcinoma was found in the cerebral cortex (A), and carcinoma cells were disseminated in the subarachnoid space (B) and the choroid plexus(C, D).

The tumor cells exhibited the same morphology as those observed in urine and CSF cytology. No primary lesions were found in the pancreatic or biliary systems. Adhesions were evident in the pelvic floor connective tissue, and direct invasion of the tumor was observed in the rectal wall, prostate, and seminal vesicles, with the loss of the layered structure in the rectal wall. Extensive involvement was seen in the retroperitoneum, including the duodenum, tissues behind the pancreas, renal hilum, perirenal area, and subserosal layer of the cecum. Osteoblastic tumors were observed in the sternum, iliac bone, femur, and thoracic vertebrae, indicative of hematogenous metastasis. Multiple lymph node metastases were found in the neck, axilla, para-aortic region, pelvic region, and inguinal lymph nodes. Numerous tumors were observed in the subarachnoid space and sulci, and tumor invasion was seen just below and within the choroid plexus (Figure 5). 

## Discussion

In a retrospective analysis of 1,514 bladder surgical specimens or biopsies at our institution from 2011 to 2019, only four cases (0.3%) exhibited dominance of PUC, and five cases (0.3%) partially contained PUC. Complications related to carcinomatous meningitis were identified in 10% of patients during the course of the disease [4]. Considering only 33 cases of carcinomatous meningitis attributed to bladder cancer were reported so far [5], the incidence of carcinomatous meningitis caused by PUC appears relatively elevated.

Only five autopsy cases of PUC have been documented to date [6-10]. In all cases, the primary tumor originated in the bladder, and all patients were male. Immunohistochemically, 80% of cases (4 out of 5) were positive for both CK7 and CK20, 100% of cases (4 out of 4) were positive for GATA3, and loss of E-cadherin staining was observed in 60% of cases (3 out of 5).

In our specific case, the CSF and serum CA19-9 levels eventually rose significantly. In our institution's investigation, we encountered a case of unresectable PUC of the urinary bladder with an elevated pre-treatment serum CA19-9 level of 3,520 U/mL. Elevated serum CA19-9 levels in bladder PUC have been reported from other facilities [7,10-11]. Immunohistochemical studies of CA19-9 have been conducted across various tissues. CA19-9 is detected in tumor tissues such as pancreatic, gastric, colorectal, gallbladder, lung, thyroid, ovarian, renal, bladder, and prostate cancers, among others. In normal tissues, CA19-9 is present in the epithelium of the ducts of the pancreas, gallbladder, stomach, bronchus, and prostate, but generally not in the kidney and bladder [12]. A case involving unilateral hydronephrosis caused by non-neoplastic ureteral stenosis exhibited elevated CA19-9 (16,000 U/mL) and SPan-1 (2,500 U/mL) levels [13]. However, hydronephrosis was not evident in our case. Immunohistochemical staining using anti-CA19-9 antibodies confirmed the positivity of tumor cells for CA19-9. Given the absence of a primary tumor other than bladder cancer and the lack of other causative factors such as hydronephrosis, the heightened serum CA19-9 levels in this case were attributed to tumor production mechanisms. In cases where carcinomatous meningitis is suspected, repeated cerebrospinal fluid cytology and creation of cell blocks for immunohistochemical analysis are useful in identifying the primary cancer site.

The increased levels of SPan-1 in the serum were contemplated as a potential molecular homology between the CA19-9 and SPan-1 antigens. Both CA19-9 and SPan-1 antibodies bind to the carbohydrate antigen sialyl Lewis a (CA19-9), and in vitro studies have demonstrated competition between the two antibodies [14]. Unfortunately, due to the unavailability of commercially accessible SPan-1 antibodies for immunostaining, we were unable to perform staining using actual tumor cells.

Moreover, hydrocephalus accompanied the carcinomatous meningitis in this case. Traditionally, communicating hydrocephalus is caused by impaired cerebrospinal fluid absorption due to obstruction of arachnoid granules caused by fibrosis and thrombus associated with inflammation [15]. However, our autopsy findings did not reveal evidence of occlusion of the arachnoid granules in the superior sagittal sinus or an apparent origin of occlusion within the ventricles. Conversely, we observed tumor cell seeding into the subarachnoid space and tumor invasion just below and within the choroid plexus. Consequently, we speculate that the hydrocephalus in this case resulted from a failure of spinal fluid circulation due to carcinomatous meningitis, and exacerbation of hydrocephalus was definitively diagnosed as the direct cause of death.

## Conclusions

This case highlights the possibility that invasive bladder cancer, such as PUC, may cause carcinomatous meningitis. PUC is a rare malignancy but should be considered as a differential diagnosis in the absence of a gastrointestinal tumor when serum CA19-9 levels are elevated. Further studies are needed to identify whether serum CA19-9 levels are a biomarker for bladder PUC.
